# Mortality and morbidity trends and predictors of mortality in under-five children with severe acute malnutrition in Hadiya zone, South Ethiopia: a four-year retrospective review of hospital-based records (2012–2015)

**DOI:** 10.1186/s40795-017-0135-5

**Published:** 2017-02-27

**Authors:** Tadele Yohannes, Tariku Laelago, Menen Ayele, Temesgen Tamrat

**Affiliations:** Hossana College of Health Sciences, Hossana, Ethiopia

**Keywords:** Severe acute malnutrition, Morbidity, Mortality, Retrospective record review

## Abstract

**Background:**

Severe acute malnutrition remains one of the most common causes of morbidity and mortality in Sub-Saharan Africa. The objective of this study was to investigate morbidity and mortality trends and factors associated with mortality of under-five children admitted and managed for severe acute malnutrition in NEMMH.

**Methods:**

Four years retrospective cohort study was conducted on 500 under-five children admitted with the diagnosis of severe acute malnutrition. The study population was all under- five children admitted to the inpatient nutrition unit between 2012 and 2015. Data was entered using Epi-Data version 3.1 and exported to SPSS version 16 for analysis. A Kaplan- Meier curve was also used to estimate survival probability of different types of severe acute malnutrition. Cox proportional hazards regression was used to predict the risk of death among predictor while adjusting for other variables. A P-value less than 0.05 was considered as statistically significant.

**Result:**

A total of 500 children were enrolled into the study. Kwashiorkor was the most frequently recorded morbidity accounting for 43.0%. Pneumonia was seen the commonest form of comorbid disease. It was the most common co-morbidity across all morbidity groups. (27.6% in kwashiorkor, 37.5% in marasmus and 37.7% in marasmic-kwashiorkor). The average length of stay in the hospital was 11 days.

Children with new admission were 86% less likely to die than repeated admission given that the children were admitted to paediatric ward (HR: 0.14, 95% CI: (0.06, 0.35). Kaplan Meier survival curves also showed children with marasmus and those with repeated admission had reduced survival rates. The overall mortality rate was 7%. The mortality trends vary irregularly in each year but morbidity trend increased with admission from 2014 to 2015.

**Conclusion:**

Mortality trends of SAM vary irregularly across the years but morbidity trends increased with admission from 2014 to 2015. An admission type was significantly associated with mortality. Morbidity and co-morbid diseases did not show significant effect on mortality of the children. Health extension workers and stakeholders should give due concern on promotion of proper nutrition in a community.

## Background

Malnutrition remains one of the most common causes of morbidity and mortality among children throughout the world. It has been responsible, directly or indirectly, for 60% of the 10.9 million deaths annually among under-five children. Over two-thirds of these deaths, which are often associated with inappropriate feeding practices, occur during the first year of life [1, 2].

Severe acute malnutrition (SAM) is characterised by wasting (marasmus), oedema (kwashiorkor), or both (marasmic kwashiorkor), and occurs mostly in children [3]. It arises as a consequence of a sudden period of food shortage and is associated with loss of a person’s body fat and wasting of their skeleton [4].

Globally, about 25 to 35 million under-five children have SAM and 13 million of these children live in sub-Saharan Africa. One million of those children die every year [5]. The death rate after admission is higher. Study conducted in Zambia showed death rate after admission as 46% [6].

In Ethiopia, malnutrition is one of the leading causes of morbidity and mortality in under-five children. The country has the second highest rate of malnutrition in Sub-Saharan Africa [7].

Among the principal cause of death in young children, 60.7% of deaths from diarrhoea, 52.3% of death from pneumonia, 44.8% of deaths from measles and 57.3% of death from malaria are attributable of malnutrition. Because of this high risk of death, many children with SAM are managed in hospitals [8].

In many health facilities of Ethiopia, the mortality rate from SAM is over 20% [9]. In view of this, federal minster of health has since 2007, implemented therapeutic Feeding Programme for the management of SAM. The therapeutic feeding programme is implemented by the minister of health in cooperation with UNICEF (United Nations International Children's Emergency Fund), and non-governmental organizations like save the children, United States and Valid international. The program is integrating the management of SAM into hospitals, health facilities and medical universities. SAM management includes two approaches: Therapeutic feeding unit (In patient care) for children with SAM and complications and; Out-patient therapeutic programme for children with uncomplicated SAM and with good appetite [10]. Despite the implementation of the programs, the level of admission with malnutrition is not lower as expected. Study conducted in Ethiopia shows that the number of children with SAM admitted into therapeutic feeding programme is increasing every month [11]. This information shows there are factors that could be a reason for increasing number of children With SAM. Although there may be multiple factors, at hospital level there may be an environment that helps to examine the trends of SAM and factors associated with mortality. Therefore, the current study tried to identify trends of mortality and morbidity and factors associated with mortality among under-five children attended Nigest Ellen Mohamed memorial hospital (NEMMH) from 2012 to 2015.

## Methods

### Settings and population

The study was conducted in NEMMH, which is located in Hossana town, southern Ethiopia. Hossana town is located 232 kilometres far from Addis Ababa, the capital city of Ethiopia. The hospital is serving about 2 million people. The total number of under-five children found in catchment area are 222,336.

The paediatrics department is one of inpatient department found in NEMMH. The department has nutrition rehabilitation unit. The nutrition rehabilitation unit admits children diagnosed SAM from Hossana town and surrounding areas. The admission criteria for infant less than 6 months or less than 3 kg being in breast-fed includes: the infant is too weak or feeble to suckle effectively (independently of his/her weight-for-length) or W/L (Weight-for-Length) less than 70% or Presence of bilateral oedema.

The admission criteria for 6 months to 59 months includes: W/H or W/L < 70% or MUAC < 110 mm with a Length > 65 cm or Presence of bilateral pitting oedema [10]. All children are managed using WHO standard guideline for Managing SAM.

The Study population was all under five children admitted to the inpatient nutrition unit between January 2012 and December 2015. Children with incomplete data were excluded from the study.

### Study design and sampling

Four years retrospective cohort study was conducted on under-five children admitted with the diagnosis of SAM from January 2012 to December 2015. The study was conducted from May to June, 2016. Total sampling method was used; where all under-five children admitted with diagnosis of SAM was considered for analysis. About 500 children were included in the study.

### Data collection and measurement

Data on variables of interest were extracted from patient charts, using a predesigned data collection form. The data collection forms were completed legibly by trained data collectors. The data collection form contains socio-demographic factors, diagnosis of SAM, co-morbid diseases, length of stay, admission type and outcomes of discharge. Patients’ chart numbers were collected from the paediatrics ward registration book. By using the chart numbers, charts were drawn by card room workers. The data collectors were trained on the requirements of the protocol and data to be collected. Completeness and legibility of each data collection form was audited at the end of each day by the principal investigators and supervisors to ensure accuracy.

In this study, the dependent variable was time to death of children with SAM while the independent variables included sex, age, length of stay, morbidity, residence, co-morbidity, and admission type.

Mortality was defined as death due to SAM and other comorbid disease while morbidity was referred to SAM. The data on SAM types (Marasmus, Kwashiorkor, and marasmic-Kwashiorkor) was reviewed from patient chart. Length of stay in the hospital was cross-checked by calculating the difference between date of admission and date at which the patient dead/discharged and corrections were made where inconsistency were found. Comorbid disease was defined as co-existence of any other disease/s with SAM. Admission type was assessed by two options: new and repeat admissions.

### Data processing and analysis

Data were entered using Epi-Data version 3.1 and exported to SPSS version 16 for analysis. Trend analysis was done by using STATA version 11. Descriptive statistics was used to summaries study variables.

Model diagnostics was done by using the maximum likelihood estimation. Cox regression model assumption of proportional hazards was checked by Kaplan-Meier hazard plots and testing an interaction of covariate with time. Multi-collinearity among independent variables was checked and showed no significance.

Kruskal Wallis was used to compare if means of “length of stay on the ward” and “age” for the different types of SAM were different. For categorical variables, chi-square was used to show association. A Kaplan-Meier curve was used to estimate survival probability of different types of SAM and admission types.

Bivariate analysis was done to identify associations between dependent and independent variables. Variables significant at P <0.05 level in the bivariate analysis were included in the final cox proportional regression analysis, to identify independent predictors of mortality. Finally, co-morbidity, morbidity groups and admission type were included in cox proportional hazards regression analysis. Cuzick, a non-parametric test was used to examine trend patterns for both morbidity and mortality. A p-value of less than 0.05 was considered as statically significant.

## Results

### Demographic and clinical characteristics of the children

A total of 500 under five children were enrolled in the study. Of these, 243 (48.6%) were females and 257 (51.4%) were males. The median age was 10 months, with an inter quartile range (IQR) of 3 to 24 months. The data also revealed that majority 412(82.4%) children were from out of Hossana while 88(17.6%) were from Hossana. The median length of stay was eleven days, with an IQR of 7 to 16. The overall mortality rate was 35(7.0%) (Table 1).

**Table 1:** Morbidity by admission type and demographic factors among under five children in NEMMH, Southern Ethiopia

Variables	Total (N and %)	Types of SAM			*P*-value*
Sex(*n* = 500)		Kwashiorkor (N and %)	Marasmus (N and %)	Marasmic-Kwashiorkor (N and %)	0.670
Male	257 (51.4)	97 (52.2)	70(50.0)	50(46.7)	
Female	243 (48.6)	89(47.8)	70(50.0)	57(53.3)	
Place of Residence(n = 500)					0.734
Out of Hossana	412(82.4)	154(82.8)	119(85.0)	87(81.3)	
Hossana	88(17.6)	32(17.2)	21(15.0)	20(18.7)	
Co-morbidity (*n* = 356)					0.588
Acute gastro enteritis (AGE)	78(21.9)	31(23.1)	23(22.1)	11(15.9)	
Diarrhoea	39(11)	19(14.2)	10(9.6)	5(7.2)	
PTB	15(4.2)	6(4.5)	5(4.8)	4(5.8)	
Pneumonia	118(33.1)	37(27.6)	39(37.5)	26(37.7)	
Others	106(29.8)	41(30.6)	27(26.0)	23(33.3)	
Admission type (*n* = 498)					0.234
New	444(89.2)	170(91.4)	124(88.6)	90(84.9)	
Repeat	54(10.8)	16(8.6)	16(11.4)	16(15.1)	

NOTE: (overall *n* = 433 was dictated by the variable morbidity)

Median age in months (IQR) for Kwashiorkor was 12(4–25), marasmus was 8(3–18) and marasmic-kwashiorkor was 12(8–36). *P* = 0.0001(Kruskal –Wallis)

*Tested using Chi square

### Patterns of morbidity

Kwashiorkor (43.0%) was the most frequently recorded morbidity followed by marasmus (32.3%). Majority (89.2%) of admission type was new. Chi square test revealed that there was no association between Childs’ sex, type of admission, residence and type of morbidity. Pneumonia was the most common co-morbidity across all morbidity groups (27.6% in kwashiorkor, 37.5% in marasmus and 37.7% in marasmic-kwashiorkor). On the other hand, Pulmonary tuberculosis (PTB) had the lowest prevalence across the co-morbidity groups (4.5% in kwashiorkor, 4.8% in marasmus and 5.8% in the marasmic-kwashiorkor) (*p* = 0.588) (Table 1).

### Patterns of co-morbidity

There were various co-morbidities that were recorded in the data. The co-morbidities included PTB, Pneumonia, AGE, acute febrile illness, anaemia, conjunctivitis, malaria, intestinal parasite, burn, candidiasis, cellulitis, congestive heart failure, diarrhoea, Down syndrome, epilepsy, Guillain–Barré syndrome, rickets, scabies, septic shock, skin lesion, nephritic syndrome, hydrocele, hypoglycaemia, and impetigo. The co-morbidities were analysed based on frequencies and the co-morbidities with the lowest frequencies (*n* < 15) were grouped together and labelled as “others”. Five co-morbidity groups’ namely AGE, Diarrhoea, PTB, Pneumonia and others were finally analysed as a single variable labelled co-morbidity. The median age for pneumonia and diarrhoea was 11 months (IQR 3–24). Age had insignificant association with co-morbidity (*P* = 0.282). Patterns of co-morbidity differed significantly across sex of children (Table 2).

**Table 2:** Co-morbidity groups by admission type and demographic factors among under five children in NEMMH, Southern Ethiopia

Variables	Total (N and %)	Co-morbidity diseases					*P*-value*
Sex (*n* = 500)		AGE (%)	Diarrhoea (%)	PTB (%)	Pneumonia (%)	Others (%)	0.043
Male	257 (51.4)	33 (42.3)	23 (59)	4 (26.7)	69 (58.5)	51 (48.1)	
Female	243 (48.6)	45 (57.7)	16 (41)	11 (73.3)	49 (41.5)	55 (51.9)	
Place of Residence (*n* = 500)							0.152
Out of Hossana	412 (82.4)	62 (79.5)	35 (89.7)	15 (100)	93 (78.8)	90 (84.9)	
Hossana	88 (17.6)	16 (20.5)	4 (10.3)	0 (0)	25 (21.2)	16 (15.1)	
Admission type (*n* = 498)							0.672
New	444 (89.2)	72 (92.3)	34 (87.2)	12 (80)	106 (89.8)	95 (89.6)	
Repeat	54 (10.8)	6 (7.7)	5 (12.8)	3 (20)	12 (10.2)	11 (10.4)	

NOTE: (overall n = 356 was dictated by the variable co-morbidity)

Median age in months(IQR) for AGE 8(2–12), diarrhea 11(3–24), PTB 12(7–36), pneumonia 11(3–24), others 9(3–24) *P* = 0.282 (Kruskal –Wallis)

*Tested using Chi square

### Length of stay in different morbidity and co-morbidity groups

The length of stay on the ward in different morbidity and co-morbidity groups did not differ significantly (*p* = 0.740 and 0.798 respectively). However, there were significant differences between admission type, with *p* = 0.016. Children with new admission type had the longest length of stay of 11 days (IQR 7–19) (Table 3).

**Table 3:** Comparison of length of stay among under five children with SAM attending NEMMH, Southern Ethiopia

Variables	Frequency	Median Length of stay in days	Inter quartile range (25–75) in days	*P-value
Morbidity (*n* = 433)				0.740
Kwashiorkor	186	12	4–25	
Marasmus	140	8	3–18	
Marasmic-Kwashiorkor	107	12	8–36	
Co-morbidity (*n* = 356)				0.798
AGE	78	8	2–12	
Diarrhoea	39	11	3–24	
PTB	15	12	7–36	
Pneumonia	118	11	3–24	
Others	106	9	3–24	
Admission type (*n* = 498)				0.016
New	444	11	7–19	
Repeat	54	8	5–14	

*Tested using Kruskal –Wallis

**Fig. 1:**
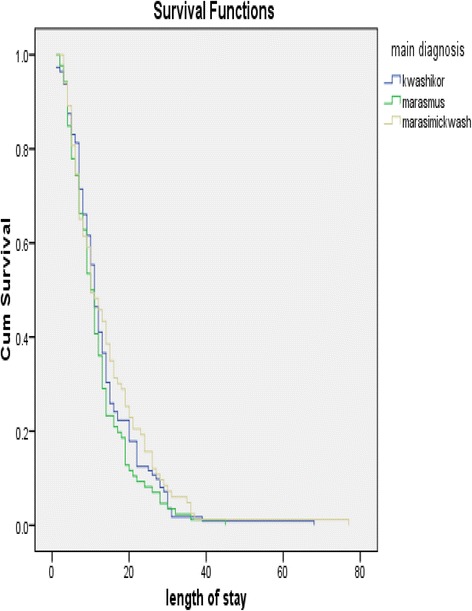
Survival estimates of child with diagnosis of SAM

**Fig. 2:**
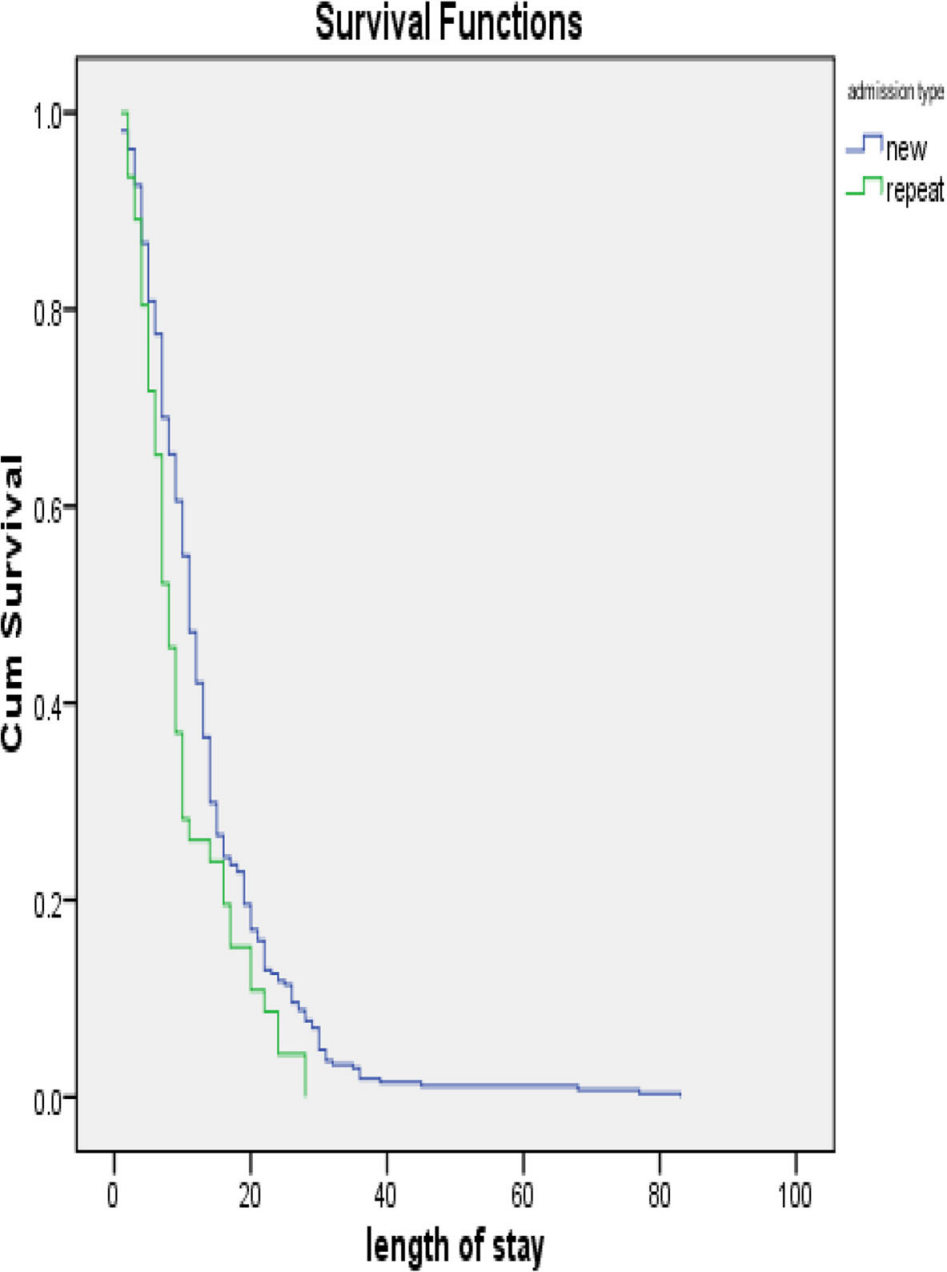
Survival estimates among children with SAM and admission type

Total person time at risk is 4132 days. The incidence rate was estimated at 0.077 per day or 28.1 per year. The different survival times were depicted using Kaplan Meier survival curves. They compare the chances of survival in the different morbidity groups given that the child was admitted to paediatric ward for a specified number of days. Children with marasmus and repeated admission had reduced survival rates given that the children come from similar area, have similar co-morbidity, sex and age group (Figs. 1 and 2).

Cox hazard regression was also used to show the instantaneous risk of death given that the child was admitted to paediatric ward. It revealed that admission type was significant (*p* < 0.001). Children with new admission were 86% less likely to die than repeated admission given that the children were admitted to paediatric ward (HR: 0.14, 95% CI: (0.06, 0.35)) (Table 4).

**Table 4:** Probability of death among under five children with SAM attending NEMMH, Southern Ethiopia

Variables	Hazard ratio (CI)	*P-value
Morbidity (*n* = 433)		
Kwashiorkor	1.055(0.364,3.058)	0.978
Marasmus	0.936(0.284,3.081)	0.922
Marasmic-Kwashiorkor	1	
Co-morbidity (*n* = 356)		
AGE	1	0.974
Diarrhoea	1.795(.298,10.810	0.523
PTB	5.899(0,947,36.755)	0.057
Pneumonia	1.639(0.361,7.446)	0.522
Others	1	
Admission type (*n* = 498)		
New	0.140(0.057,0.348)	0.001
Repeat	1	

*Tested by using chi-square

### Morbidity and mortality trends

**Fig. 3:**
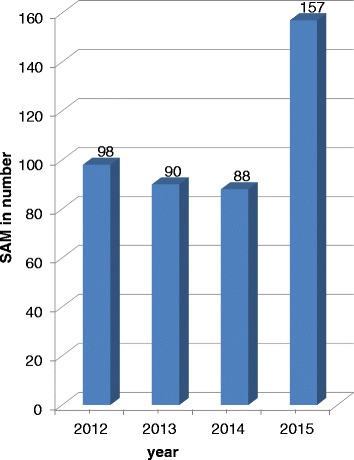
Morbidity trends of SAM from 2012–2015

**Fig. 4:**
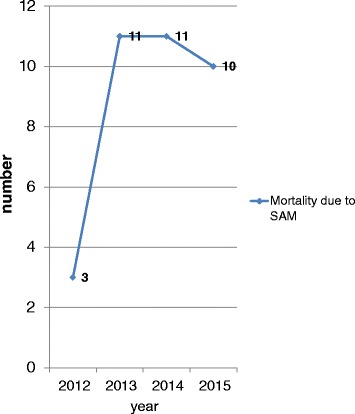
Mortality trends from 2012–2015

Generally, mortality trends vary irregularly in each year but morbidity trends decreased from 2012 to 2014 and increased from 2014 to 2015 (Figs. 3 and 4). Cuzick, a non-parametric test for trends was used to show the significance of the trends. The trends for morbidity were significant (*P* = 0.008) but the trends for mortality were insignificant (*P* = 0.851).

## Discussion

This study was conducted on 500 severely malnourished children admitted to Nigest Elleni Mohammad memorial hospital from January 2012 to December 2015 to investigate morbidity and mortality trends and factors associated with mortality of children with SAM.

The findings of the study showed that, mortality trends vary irregularly in each year, but morbidity trend decreased with admission from 2012 to 2014 and increased from 2014 to 2015. The morbidity trends in current study are in agreement with 2015 national report of Uncief. The report showed that, the number of children 6 months to 59 months with SAM admitted into therapeutic feeding programme is increasing every month. The south Ethiopia region has high trend of admission. The increase in admissions is attributed to the failure of the autumn rains that has greatly contributed to the deterioration of the food security in this region [11].

Kwashiorkor was seen as the most frequently recorded type of SAM and had highest presence of comorbid diseases. This is similar with some previous studies [6, 12, 13], but it is inconsistent with study conducted in Colombia [14]. The difference may be due to difference in causes of malnutrition and life styles in the regions.

This study showed no significant association of SAM and comorbid diseases. This is inconsistent with study done in south Ethiopia, which showed morbidity of the child by diarrhoea was associated with SAM [15]. The difference may be due to difference in study design.

Pneumonia was seen the commonest form of comorbid disease. This may be due to inhalation of part of the diet is a common cause of pneumonia in all malnourished patients. Patients should be closely observed whilst they are being fed by the caretaker to ensure that the correct technique is being used [9].

Children with new admission were 86% less likely to die than repeated admission. This may be due to deterioration of diseases in repeated admission, as a result the chance to recover may decreases.

The death rate in current study was 7%. This finding is consistent with the minimum international standards set for management of SAM which was less than 10%. It is also less than the death rate recorded in Jimma University specialized hospital, which was 9.3% [9, 13]. The difference may due to difference in patients load. But the death rate of this study is higher than the target set by federal minster of health, which showed less than 5% [16].

In current study, the average length of stay in the hospital was 11 days; this is consistent with the minimum international standard set for management of severe acute malnutrition, which showed average length of stay less than 30 days [9]. Study conducted in Jimma university specialized hospital and South Ethiopia showed longer average length of stay than current study [12, 13]. The may be due to difference in co-morbid diseases seen among the children.

Co-morbid diseases did not show significant effect on mortality of the children. This is differing from study done in Jimma university specialized hospital, which showed children with comorbidities were more likely to die than without comorbidities. This difference may be due to small sample sizes of current study [13].

This study has the following limitations:

Because of incompleteness, the HIV status has not been included.

The study did not consider biochemical findings and medical supplies that might have influenced outcomes.

The proportion of missing value on morbidity types is big.

Further studies should be done considering multi-factorial aspects including biochemical factors and medical supplies. Studies should also be done in the hospital including HIV status with strict follow up of recording HIV status of the under five children with SAM.

## Conclusion

Mortality trends of SAM vary irregularly across the years but morbidity trends increased with admission from 2014 to 2015. An admission type was significantly associated with mortality. Morbidity and co-morbid diseases did not show significant effect on mortality of the children.

Health extension workers should promote good feeding practices and strong family planning service in families. The six strategies that have been found to promote proper nutrition in a community should be implemented by all stakeholders: Basic education, Healthy environment, Maternal and child care, Healthy social and family life, Proper agriculture and Public health measures.

## Abbreviations

AGE: Acute gastro enteritis
CMAM: Community management of acute malnutrition
HIV: Human immune deficiency virus
IQR: Inter-quartile range
NEMMH: Nigest Ellen Mohamed memorial hospital
PTB: Pulmonary tuberculosis
SAM: Severe acute malnutrition
UNICEF: United Nations International Children's Emergency Fund
WHO: World health organization.
